# Influence of semicircular canal morphology on the VOR and swimming activity in larval amphibians: a comparative study in *Xenopus* and axolotl

**DOI:** 10.3389/fneur.2025.1564585

**Published:** 2025-05-19

**Authors:** Parthena Schneider-Soupiadis, Michael Forsthofer, Felix Schneider-Soupiadis, Gilles Courtand, Rosario Sanchez-Gonzalez, François M. Lambert, Hans Straka

**Affiliations:** ^1^Faculty of Biology, Ludwig-Maximilians-University, Munich, Germany; ^2^Graduate School of Systemic Neurosciences, Ludwig-Maximilians-University, Munich, Germany; ^3^School of Life Sciences, University of Sussex, Brighton, United Kingdom; ^4^Institut de Neurosciences Cognitives et Intégratives d’Aquitaine (INCIA), CNRS UMR 5287, Université de Bordeaux, Bordeaux, France

**Keywords:** vestibulo-ocular reflex, optokinetic reflex, semicircular canal, eye movements, locomotion, *Xenopus*, axolotl

## Abstract

Gaze stabilization and locomotion rely often on an accurate sensory detection of head movements by the vestibular system. A functional relationship between vestibular sensitivity, locomotor skills and semicircular canal morphology has been suspected in vertebrates as an adaptation to eco-physiological and species-specific needs, but has only partially and indirectly documented. However, evaluating the vestibulo-ocular reflexive activity and the locomotor efficiency simultaneously with the rotational sensor geometry remains absent from the literature. From such a perspective, this study attempted to provide a simultaneous quantification of the vestibulo-ocular response, the swimming efficiency and the canal morphology in the salamander axolotl and the frog *Xenopus laevis*, two amphibian species with comparable lifestyle and identical locomotor and vestibular systems at larval stages. Animals were studied at an equivalent developmental period: the late pre-metamorphic stage where the hindlimbs start to differentiate. Larval axolotl demonstrated an angular vestibulo-ocular reflex (aVOR) with a gain ~83% lower than *Xenopus*. Like in *Xenopus* at earlier stages, the aVOR gain increased in axolotl indicating a later functional onset. The morphological comparison of the semicircular canals of both species revealed that the horizontal canal in axolotl was thinner, less curved and less coplanar to the horizontal plane compared to *Xenopus*. Additionally, the ampulla of *Xenopus* was rounder and less elongated than in axolotl. All these parameters are critical for endolymph flow and consequently for the capacity of semicircular canals to perceive head motion. Interestingly, axolotl demonstrated a reduced swimming activity, more episodic than *Xenopus*, resulting in less frequent exposure to important head accelerations. Altogether, our results provide correlative evidences for a clear functional link between semicircular canal morphology, vestibular sensitivity, influencing aVOR performance, but also locomotor capacity in two comparable species, representative of anuran and salamander amphibians. This study, even preliminary, should open the pathway for further and more complete demonstrations of this functional relationship, that seems to be commonly shared during the evolution.

## Introduction

Animal locomotion is often accompanied by reflexive gaze-stabilizing eye movements that minimize the deteriorating consequences of head motion on retinal image stability (1). Vestibulo-ocular (VOR) reflexes are known to participate predominantly in this gaze stabilization. Vestibular endorgans and neuronal circuits have been evolutionarily well conserved over the last 500 million years (2–4). Nevertheless, the vestibular system maintains a dynamic range of plasticity to respond to changing environments and demands. This adaptability has been studied in a variety of vertebrates and was found to exhibit a number of species-specific and/or eco-physiological adaptations (5). However, a basic biomechanistic rule expressed in many vertebrate species, suggests that labyrinth endorgan morphology (i.e., canal diameter, flatness) affects the endolymph flow characteristics—the movement of which is what causes the detection of motion. Therefore, labyrinth morphology can directly affect the sensitivity of vestibular rotation sensors [(6–13); for review see Lambert and Bacqué-Cazenave (14)]. As a direct physiological consequence, a robust angular VOR relies on sufficiently sized semicircular canals, which during the ontogeny of small fish and amphibian larvae often represents a critical issue and thus determines the onset and subsequent performance of the reflex (15, 16). Through this, labyrinth geometry is believed to indirectly affect locomotor capacity—which in many sighted vertebrates relies on maintaining a stable gaze. As a general tendency, it appears that animals with fast and agile locomotion more likely exhibit large semicircular canals with elongated ducts whereas smaller ducts are more common for slower locomotor regimes (17). Various studies have tried to demonstrate a phylogenetic link in vertebrate evolution between locomotor style/capacity, semicircular canal dimensions, and behavioral repertoire of a particular species (17–28). But deciphering this functional relationship remains a challenging task, primarily due to the difficulty of identifying comparable species with similar developmental patterns, locomotor style, and vestibular systems that are easily experimentally accessible. Moreover, the difficult accessibility of the inner ear, typically situated within the head and covered by bone in most vertebrates, complicates morphological comparisons, and accordingly there are very limited studies able to compare conjointly semicircular canal morphology, vestibulo-ocular motor output and the locomotor activity between two species.

The present study attempted to address this challenge by evaluating the vestibular sensitivity through the vestibulo-ocular reflex, the semicircular canal morphology, and the locomotor activity in two amphibian species: the toad *Xenopus laevis* and the salamander axolotl (*Ambystoma mexicanum*). Both species exhibit a comparable developmental pattern until mid-larval stages, have transparent tissue with the inner ear being visually exposed, and utilize undulatory tail-based swimming during their aquatic larval stages. Based on literature, animals of both species were chosen at a comparable developmental stage, where they are similar in size, display similar states of fore- and hindlimb development, and show similar visual behaviors (29). *In vitro* preparations were used to address gaze stabilization capacities during passive head motion in both amphibian larvae while locomotor performances were recorded from free swimming animals. Herein, the well-studied VOR dynamics of the behavioral performance of *Xenopus laevis* larvae (16, 29–32) was compared to the respective profiles of these gaze-stabilizing reflexes in axolotl. In parallel, the morphology of the horizontal canal was quantified using confocal microscopy. By combining these approaches, we provide cumulative evidence that suggests a link between morphology of the inner ear sensor responsible for the detection of head rotations in the horizontal plane, vestibular sensitivity, and ultimately locomotion.

## Materials and methods

### Experimental model and subject details

Behavioral and anatomical experiments were conducted on *Xenopus laevis* tadpoles (*n* = 85) and axolotl (*Ambystoma mexicanum*) larvae (*n* = 67) of either sex at developmental stages 48–56. Developmental stages were determined based on the description by Nieuwkoop and Faber (33) for *Xenopus* and by Nye et al. (34) for axolotl. Axolotl larvae were obtained from the in-house breeding facility at the Biocenter-Martinsried of the Ludwig-Maximilians-University Munich (LMU) while *Xenopus* embryos were obtained from the Biomedical Center of the LMU and transferred to the in-house animal facility. Larvae (around 20 individuals/tank) of both species were maintained in separate tanks (50-60 L) with filtered water (17–19°C) at a 12 h/12 h light/dark cycle. Animals were used randomly in experiments, when they reached the desired developmental stage. All experiments were performed in compliance with the “Principles of animal care” publication No. 86–23, revised 1985, of the National Institutes of Health and were carried out in accordance with the ARRIVE guidelines and regulations. Permission for the experiments was granted by the government of Upper Bavaria (Regierung von Oberbayern) under the license codes ROB-55.2.2532.Vet_03-17-24 and ROB-55.2.2532.Vet_02-22-54. In addition, all experiments were performed in accordance with the relevant guidelines and regulations of the LMU Munich.

### Video tracking and analysis of swimming/locomotor behaviors

**Figure 1:**
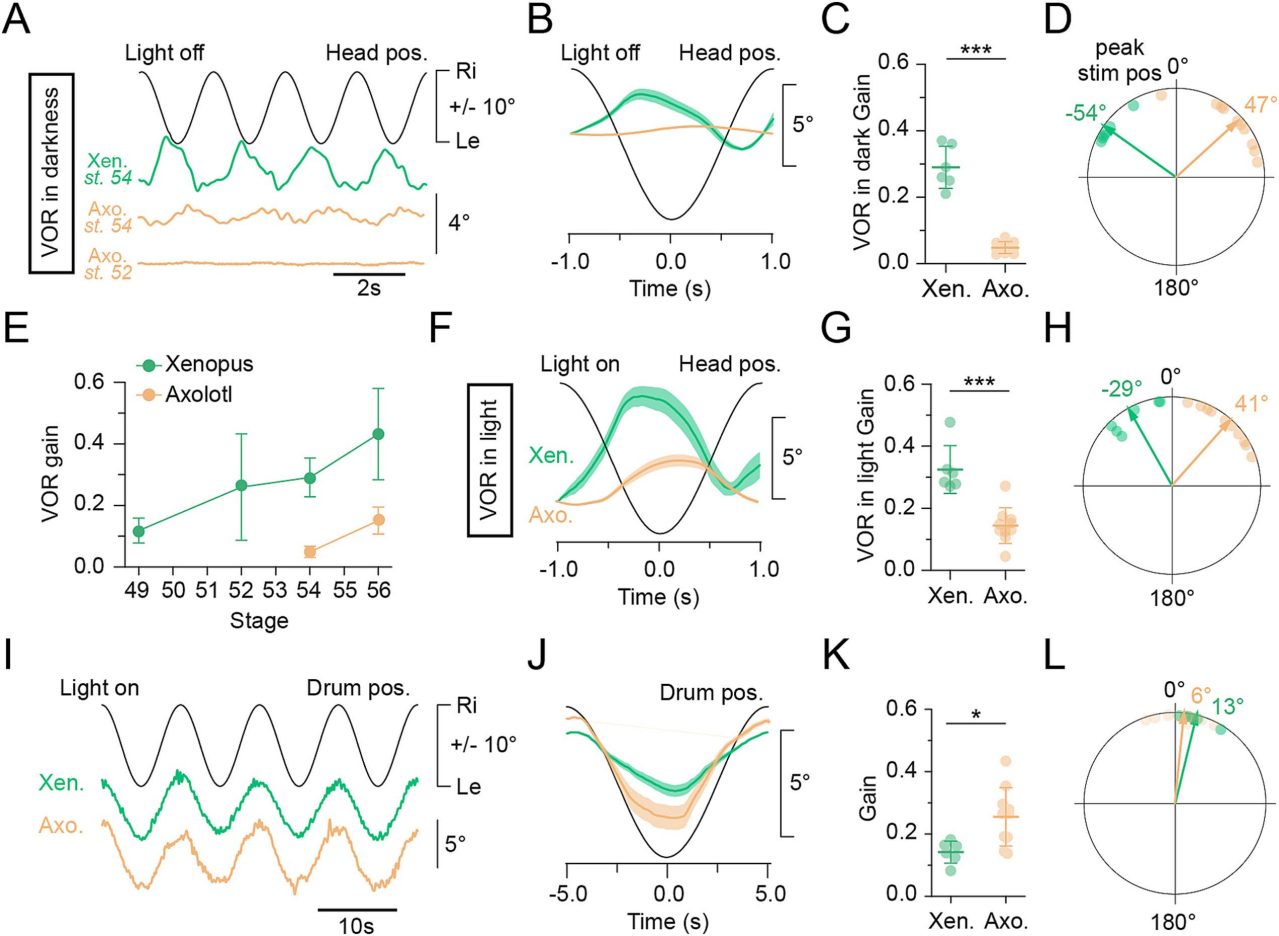
VOR and OKR eye movements. **(A–H)** Reflexive eye movements evoked by the angular vestibulo-ocular reflex (aVOR) in response to horizontal head rotation in the dark **(A–E)** and in light **(F–H)**. **(A)** Representative example of compensatory eye movements evoked by the aVOR during horizontal sinusoidal rotation of the head/table (0.5 Hz; ± 31.4°/s). Data shown in this panel **B–D**, **F–H** and **I–L** compare *Xenopus* and axolotl at stage 54. **(B)** Average response (6–15 cycles averaged) of the aVOR-driven eye movements over a single head motion cycle in larval stage 54 *Xenopus* (green) and axolotl (orange), black sine wave indicates stimulus position (head pos.; 0.5 Hz; ± 31.4°/s). **(C)** Average gain (eye motion amplitude/stimulus amplitude; mean ± SD) of the aVOR at 0.5 Hz (± 31.4°/s) *Xenopus* (*N* = 6) and axolotl (*N* = 10) respectively. ***p < 0.001; Mann-Whitney *U*-test. **(D)** Polar plots illustrating aVOR phase relations to peak stimulus position (0°, peak stim pos) from 0° to ± 180°; arrows indicate the mean vector for *Xenopus* (green) and axolotl (orange). **(E)** Averaged (±SD) gain of the aVOR through larval stages between *Xenopus* and axolotl. **(F)** Average response of the aVOR over a single head motion cycle (10 cycles averaged), black sine wave indicates stimulus position (head pos.; 0.5 Hz; ± 31.4°/s). **(G)** Average gain (eye motion amplitude/stimulus amplitude; mean ± SD) for aVOR at 0.5 Hz; ± 31.4°/s for *Xenopus* (*N* = 6) and Axolotl (*N* = 10,) respectively. ***p < 0.001; Mann-Whitney *U*-test. **(H)** Polar plots illustrating aVOR phase relations to peak stimulus position (0°, peak stim pos) from 0° to ± 180°; arrows indicate the mean vector for *Xenopus* (green) and axolotl (orange). **(I)** Representative reflexive eye movements evoked by the optokinetic reflex (OKR) during horizontal sinusoidal rotation of a black and white striped pattern (0.1 Hz; ± 31.4°/s). **(J)** Average response of the OKR over a single head motion cycle (10 cycles averaged), black sine wave indicates stimulus position (virtual drum pos.; 0.1 Hz; ± 6.28°/s). **(K)** Average gain (eye motion amplitude/stimulus amplitude; mean ± SD) of the OKR at 0.1 Hz; ± 6.28°/s for *Xenopus* (*N* = 6) and axolotl (*N* = 10,) respectively. ***p < 0.001; Mann-Whitney *U*-test. **(L)** Polar plots illustrating OKR phase relations to peak stimulus position (0°, peak stim pos) from 0° to ± 180°; arrows indicate the mean vector for *Xenopus* (green) and axolotl (orange).

Freely swimming animals were filmed to extract and quantify locomotor kinematic parameters in *Xenopus* (n = 13) and axolotl (n = 12) larvae. Animals were placed in a circular petri dish (one animal at a time; diameter 18.8 cm, water height ~2 cm; Figure 1A) and illuminated from below with an illumination box (Kaiser 2,450 slimlite LED). Animals were video-recorded from above with a color camera (Basler ace, acA1300-200uc, 106754), mounted on a tripod (Manfrotto 290 xtra, MH804-3 W) using pylon viewer (5.0.12.11830, Basler). Videos were recorded for 1 min at a framerate of 30 FPS with a resolution of 1,200 × 1,200 pixels and were stored in the avi-file format. Recorded sequences, or swim bouts (defined as a continuous and uninterrupted locomotor event), consisted of either spontaneous swimming or were induced by a gentle water flow produced with a plastic pipette at the tip of tail.

**Figure 2:**
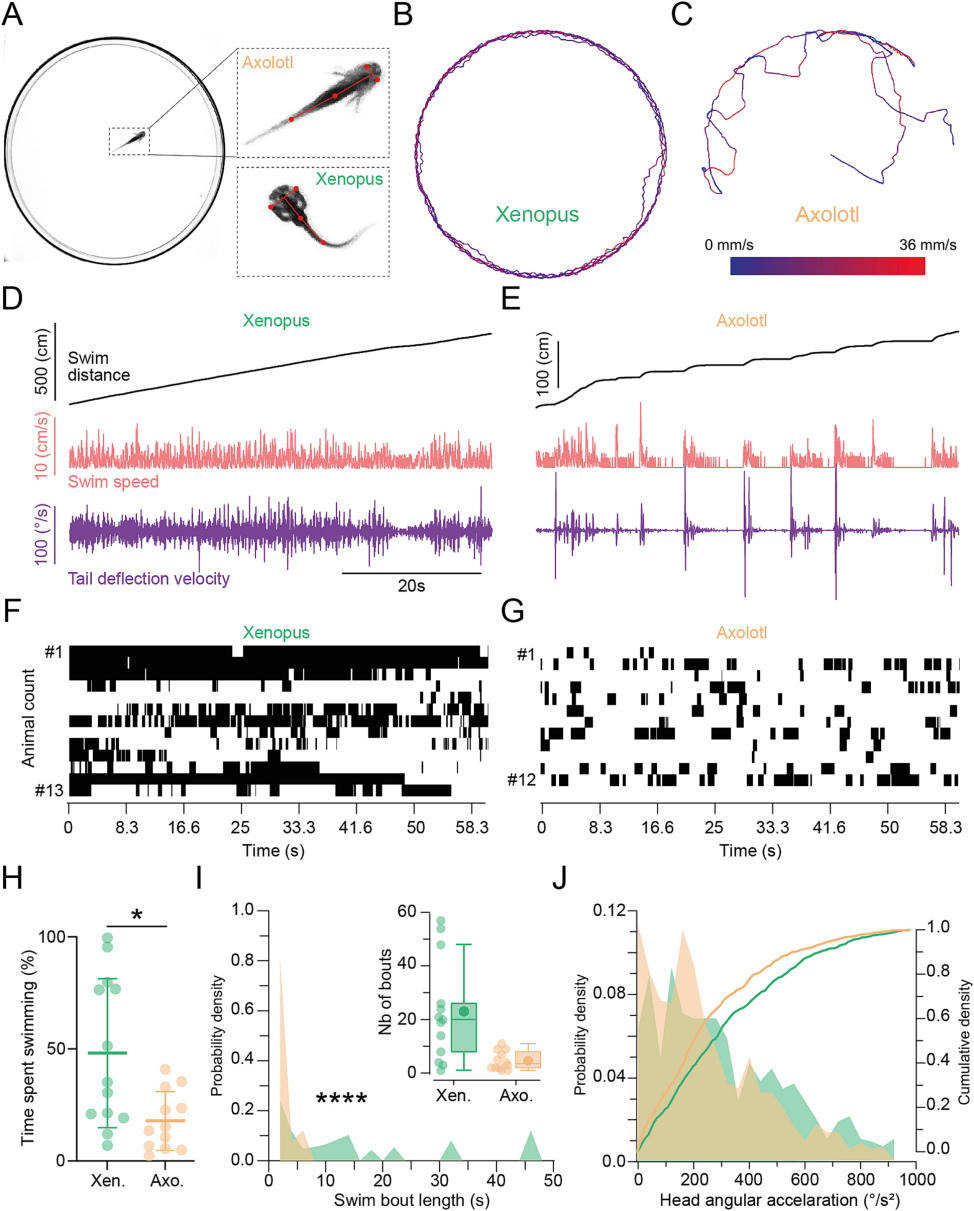
Locomotor kinematics and patterns in stage 54 *Xenopus* and axolotl. **(A)** Example of an axolotl in the recording chamber. Magnification on the right side shows the skeleton fitted on the body of *Xenopus* (Xen.) and axolotl (axo.) to swim trajectories and kinematics. **(B,C)** Representative recording sessions showing swim trajectories, color-coded for velocity, for *Xenopus* **(B)** and axolotl **(C)** during free swimming. **(D,E)** Representative examples (corresponding to recording sessions shown in **B** and **C**) of the overall swim distance, swim speed, and tail deflection velocity in *Xenopus* **(D)** and axolotl **(E)** respectively. **(F,G)** Swimming event count for each individual animal represented in an event map, over the whole recording session, indicating periods of active locomotion in black. Each horizontal line represents the number of swim bouts for a given tadpole. **(H)** Percentage of time spent swimming over the recording session plotted as mean ± SD, *p* = 0.0188, Mann Whitney U-test, two-tailed. **(I)** Probability density of time-weighted swim bout lengths illustrating the contribution of individual bouts to swim duration, *p* < 0.0001, Kolmogorov–Smirnov test. Inset shows absolute number of bouts for each species. **(J)** Probability density (left Y axis) and cumulative density (right Y axis) of angular head accelerations in *Xenopus* (green) and axolotl (orange) during swimming, *p =* 0.012, Kolmogorov–Smirnov test.

Video recordings were analyzed offline. The video files were converted into mp4-file formats using ffmpeg (35), and the spatial position of the animals was extracted using the SLEAP framework (36). In brief, a single-animal deep-learning model was trained on 200 manually labelled frames for each species (Backbone: UNet, Epochs: 200, Plateau: 10, Batch size: 5). After verifying the quality of labelling, the model was used to identify external anatomical landmarks of the animal comprising the eyes, center of the skull, and body center of mass as well as 2 tail points (see Figure 2A). This was subsequently used to infer the position of the landmarks across different videos. Videos of the motion-tracked animals were exported with the respective markers for visualization purposes. Marker coordinates were exported as time series in the hdf5-format and were further analyzed in Python (python 3.7) using the Spyder 4 IDE. The position of the body center of mass over time was plotted for visualization of the swim path, and to calculate the traveled distance between consecutive frames. The differentiation of this yielded the swim velocity of animals, and the integral the total swim distance within a recording. Finally, the angle between the brain-center of mass axis and the center of mass—tail axis was calculated and differentiated to calculate the tail deflection velocity. Swim and tail velocity traces were then smoothed with a Savitzky–Golay filter (window length 15, order 3). Active swim bouts were identified by finding periods where animals crossed a velocity threshold (3 mm/s) and a tail velocity threshold (3°/s) (i.e., the animal moved its tail while moving forward, eliminating either tail movements without locomotion, or passive horizontal motions). The duration of such swimming events was then used to calculate the time animals spent actively locomoting. For the calculation of swim kinematics, the angle of the line between the left eye and right eye was measured relative to the horizontal axis of the video and was differentiated twice to obtain the angular acceleration of the head during swimming.

For statistical comparison of swim kinematics in Python, head angular acceleration and swim velocity during active swim episodes were pooled across animals within each species and plotted as probability density histograms. Data were tested for belonging to the same distribution with a Kolmogorov–Smirnov test (scipy toolbox). To compare the contribution of swim bouts to overall swimming, rather than mere occurrence of bouts, swim bouts were weighted by their duration, so that a 30s swim bout would contribute 30x the counts of a histogram as a 1 s bout. This pooled array was then plotted as a histogram of the probability density per species, and the two distributions were compared with a Kolmogorov–Smirnov test. Additionally, swim bouts were visualized as a heatmap displaying active swimming as black bars (pyplot toolbox).

### Experimental preparation for eye and tail tracking

Semi-intact preparations for *in vitro* eye motion tracking experiments were performed according to a previously described protocol (30, 37). Accordingly, axolotl and *Xenopus* larvae were deeply anesthetized in 0.05% 3-aminobenzoic acid ethyl ester methanesulfonate at room temperature (MS-222; Pharmaq Ltd. UK) for 3 min (38), transferred to a petri dish (Ø 5 cm) containing ice-cold Ringer solution (75 mM NaCl, 25 mM NaHCO_3_, 2 mM CaCl_2_, 2 mM KCl, 0.1 mM MgCl_2_, and 11 mM glucose, pH 7.4) and decapitated at experiment-dependent levels of the spinal cord. Following removal of the lower jaws and visceral organs, the head was mechanically secured dorsal side-up with insect pins (0.2 mm, Fine Science Tools) onto the Sylgard-lined floor of the petri dish. Thereafter, the skin directly above the skull and bilateral otic capsules was taken off and the cartilaginous tissue until the first 5–8 spinal segments was opened. The forebrain was disconnected and the choroid plexus covering the fourth ventricle was removed to allow access of the Ringer solution. The remaining central nervous system, visual, and vestibular sensory periphery with afferent connections, and extraocular motor nerves remained functionally preserved. This allowed prolonged recordings of robust eye movements during application of visual and vestibular motion stimuli and spontaneous tail undulations (30–32). After the surgery, all preparations were allowed to recover for ~1–3 h at 17°C before commencing with the recording session. During a recording session, preparations were mechanically secured in the center of the Sylgard-lined recording chamber (Ø 5 cm) and were continuously supplied with oxygenated (Carbogen: 95% O_2_, 5% CO_2_) Ringer solution at a constant temperature of 17.5 ± 0.5°C.

### Visual and vestibular motion stimulation, recording and analysis

The preserved neuronal innervation of all extraocular muscles in semi-intact preparations allowed activation and video-recording of eye movements in response to vestibular and visual motion stimulation. In order to comply with the European and national “3R” regulation (Refine, Replace and Re-use), OKR and VOR measurements from larval axolotl (*n* = 10 at stage 54; *n* = 5 at stage 56) were compared to data from larval *Xenopus* previously obtained in the same experimental conditions in (30, 32) (*n* = 6 at stage 54, *n* = 4 at stage 56). Activation of the vestibular endorgans was performed with a six degrees of freedom motion stimulator (PI H-840, Physik Instrumente, Karlsruhe, Germany). Motion stimuli consisted of sinusoidal horizontal rotations at 0.5 Hz and a positional excursion of ±10° with a peak rotational velocity of ±31.4°/s. Large-field visual pattern motion was provided in an open-loop virtual reality setting consisting of an open cylindrical screen surrounding the recording chamber horizontally by encompassing 275° of the visual field with a diameter of 8 cm and a height of 5 cm (29, 32, 39). Three digital light processing (DLP) video projectors (Aiptek V60, Apitek International GmbH, Willich, Germany), installed in 90° angles to each other were affixed around the motion platform and projected a visual pattern at a refresh rate of 60 Hz onto the screen. The pattern consisted of equally spaced vertical, black and white stripes with a spatial size of 16°/16°. The horizontal pattern motion consisted of sinusoidal oscillations at 0.1 Hz and positional excursions of ±10° (±6.28°/s peak velocity). For all experiments, the Sylgard-lined recording chamber with the affixed preparation was centered inside the cylindrical screen that co-aligned with the vertical rotation axis of the vestibular motion stimulator. Visuo-vestibular motion stimuli were applied separately to evoke a VOR in darkness, VOR in light (in the presence of the world-stationary vertical black and white stripes) or an OKR response.

The movements of both eyes were captured non-invasively with a camera (Grasshopper 0.3 MP Mono Fire-Wire 1394b, PointGrey, Vancouver, BC, Canada) equipped with an objective lens (Optem Zoom 70XL, Qioptiq Photonics GmbH & Co. KG, Germany; M25 x 0.75 + 0.25) and infrared-filter (800 nm long pass) at a frame rate of 30 FPS [see Soupiadou et al. (32); Forsthofer and Straka (39) for details], while the preparation was illuminated from above by an infrared light source (840 nm). Eye position was extracted in real time from the video by automated fitting of an ellipse around each eye (29). The angle between the major axis of each ellipse and the vertical image axis was calculated in a frame-by-frame manner by a custom-written software (15) and was recorded and stored for off-line analysis along with the visual/vestibular motion stimulus (Spike2 version 7.04, Cambridge Electronic Design Ltd.).

Visuo-vestibular evoked eye motion data were acquired in Spike2, subsequently exported in matlab files (The MathWorks Inc.) and analyzed off-line with custom-written Python 3 scripts. Prior to this procedure, stimulus and eye motion recordings were resampled at 200 Hz and filtered with a 4 Hz low-pass Butterworth filter as implemented previously (39, 40). For better visualization of species-specific differences, left and right eye conjugated positions were averaged (40, 41). Eye movement and corresponding stimulus profiles were further segmented into individual stimulus cycles from peak-to-peak positions, and averaged across multiple, uninterrupted (15–20) cycles within each animal. Sporadic cycles with either stimulus-evoked resetting fast-phases or spontaneous jerking movements were manually identified and excluded from further analysis. Eye movements were quantitatively assessed by calculating the response gain (peak-to-peak eye position/peak-to-peak stimulus position) and by determining the phase relation of the motion-induced eye movements with respect to the stimulus position. Phase calculations were obtained by comparing the timing of the average response peak with the timing of the maximal table position or visual motion pattern excursion. Phase relationship were assessed by circular plot analysis (40). The “r” value given in the results section is the strength of the mean vector and indicates the strength of the coupling.

### Locomotion-induced eye movements, recording and analysis

Horizontal eye movements were recorded during spontaneous swimming episodes in head-fixed *in vitro* semi-intact preparations with intact tails. Like for the OKR/VOR measurements, locomotor-induced ocular activity from larval axolotl (*n* = 7 at stage 53–54) were compared to data from larval *Xenopus* previously obtained in the same experimental conditions in Bacqué-Cazenave et al. (30) (*n* = 7 at stage 53–54), in accordance with the 3R regulation. Both optic nerves were transected and the head was firmly secured to the Sylgard floor to exclude any visual and vestibular sensory input during the recording session. Movements of the eyes and tail were recorded at 250 frames per second with a high-speed digital camera (Basler ace, acA1300-200uc, 106754) equipped with a micro-lens (Optem MVZL macro video zoom lens, QIOPTIQ). The camera was placed above the center of the preparations and videos were recorded at a shutter speed of 3,000 μs and a resolution of 1,200 × 800 px, stored in an avi-file format. The position of both eyes and the tail was offline analyzed using an automated, custom-written software in Python 3.5 (Animotion collaborative core facility, INCIA CNRS UMR5287, Université de Bordeaux, http://www.incia.ubordeaux1.fr/spip.php?rubrique193). Angles between the major axis of the elliptically shaped eyes as well as the angles of the positional deviation of the first five tail myotomes relative to the longitudinal head axis were calculated frame-by-frame.

### Semicircular 2D and 3D morphological measurements

The dimensions of the horizontal semicircular canals were determined in a subset of semi-intact preparations of axolotl (stage 54, *n* = 8; stage 56, *n* = 3) and *Xenopus* larvae (stage 54, *n* = 8; stage 56, *n* = 3). Each specimen was mechanically secured to a Sylgard-lined Petri dish (Ø 5 cm), carefully cleaned from connective and muscle tissue attached to the exterior of the inner ear capsule and photographed with bright-field illumination using a camera (Axiocam 305 color, Carl Zeiss Microscopy GmbH) mounted onto a stereoscope (SteREO Discovery.V20, Carl Zeiss Microscopy GmbH).

To visualize and analyze the morphology of the horizontal semicircular canal within the inner ear compartment, a small volume of dextran conjugated tetramethylrhodamine (10.000 MW; Invitrogen, D1817) was injected into the endolymphatic compartment. Microelectrodes for the injections were fabricated from borosilicate glass (diameter: 1.5 mm GB150-8P, Science Products, Hofheim, Germany) with a horizontal puller (Sutter Instrument, P-87 Brown/ Flaming). Thereafter, tips were broken under visual control and beveled (Micropipette Grinder EG-45, Narishige) to a diameter of ~30 μm (30° angle). Microelectrodes were filled with a 20% solution of the fluorescent dye, inserted into an electrode holder connected to a pressure injection device (PDES-01 AM, npi electronic GmbH, Tamm, Germany) and mounted onto a 3-axis micromanipulator (Bachhofer, Reutlingen). Microelectrodes were inserted into the endolymphatic cavity of the *common crus*, where the anterior and posterior vertical semicircular canals merge dorso-medially [see Miller Bever et al. (42)]. The fluorescent dye (~0.5 μL) was injected with 2–6 pressure pulses of 1 bar and 100 ms duration over a period of ~5 min followed by a period of ~2 h to allow the fluorescent dye to spread and label the entire endolymphatic space. Fluorescent images of the inner ear were captured on a stereomicroscope (SteReo Lumar.V12, NeoLumar S 0.8x objective equipped with an AxioCam MRm camera, Carl Zeiss Microscopy GmbH) to quantify the endolymphatic lumen- and circuit radius $\left(\text{circuit radius}=\sqrt{\left[\frac{Ra^{2}+Rb^{2}}{2}\right]}\right)$ of each semicircular canal (ZEN lite, CZI, Zeiss, Germany).

In an additional set of animals, 30 min post injection, preparations were mounted in PBS using a custom metal spacer for confocal imaging to 3D reconstruct the structure of the horizontal semicircular canal. Images were taken at the Core Bioimaging Facility of the Biomedical Center of the LMU on a Leica SP8 upright microscope, using solid state laser excitation at 552 nm. Images were acquired with a 10x objective (HC PL FLUOTAR, 10x/0.30; WD 11 mm, dry), image pixel size was 1.47 μm. TMR fluorescent images were recorded with external, non-descanned hybrid detectors (HyDs) and recording was sequentially to avoid bleed-through. 3D analysis of the imaged horizontal canals was performed in Fiji (43). First a region of interest (ROI) was manually selected around the horizontal canal, cropped, and smoothed with a Gaussian blur filter (filter value = 4; indicates the number of pixels averaged in the neighborhood of a given pixel). Then the image threshold was adjusted and a new black and white stack created. For the 3D measurements, the 3D manager plugin was used (44). After 3D segmentation was applied, the volume, elongation ratio, and flatness of the canal was measured. For the measurement of canal cross section areas, the thresholded images were imported from Fiji in the 3DSlicer 5.2 (45). A center line within the canal was defined using the Vascular Modeling Toolkit^1^ and from this diameter values were measured from one end of the canal to the other.

### Principal component analysis of canal morphology, gaze stabilization and swim parameters

To extract features from canal cross-section measurements, all cross-section profiles from all animals were interpolated to the same length and centered around the mean, and subsequently treated as the original feature vectors. The first 5 principal components of these vectors were used as the canal cross-section features. To leverage the independent samples of different measures across different cohorts of animals for compiling descriptive summary statistics, we employed a resampling strategy with a dimensionality reduction technique for visualization. Assuming that the measurements of each variable were representative of the underlying distribution, we resampled the data from each variable to generate semi-synthetic data points for all other cohorts of animals for which the respective measurement was missing, thereby creating a comprehensive semi-synthetic dataset of full feature vectors for each animal. Importantly this is the most conservative approach, since it assumes that the variables for each animal are independently drawn from their respective distributions. This resampling procedure was repeated 10,000 times to gain a robust estimate of the underlying sampling distribution. Each measurement type was subsequently normalized and centered around zero. We then computed the average principal components of these resampled datasets. A most representative resampled dataset was chosen by finding the minimum difference to the average resampled dataset. The results of this PCA analysis were used to illustrate the relative strengths of correlative relationships in the data. Subsequently the measurement vectors of each animal of this most representative dataset were projected onto the average principal components by computing the dot product. The entire analysis was performed with custom written python code (Python 3) and third-party python packages (numpy, scipy).

### Statistics and software

Statistical analysis and individual plots were performed in Prism 9 (Graph-Pad Software Inc. United States) or Python 3.7. Data were plotted as column scatter plots with mean ± standard deviation or standard error of the mean (SD; SEM). Statistical differences between experimental groups were calculated with the non-parametric Mann–Whitney *U*-test for unpaired parameters, the Wilcoxon signed-rank test for paired parameters, and the Kruskal-Wallis-test and a Dunn’s test (unpaired parameters) for multiple comparisons and indicated as *p*-values (* *p* < 0.05; ** *p* < 0.001; *** *p* < 0.0001). Circular statistics for phase relationships of eye movements were calculated in Oriana (Version 4; Kovach Computing Services). A mean vector was computed from phase values, providing both the mean direction and the vector’s strength, serving as an indicator of data clustering on a scale from 0 to 1. Differences in phase values were identified with a Watson-Williams-F test. Figures were compiled in Affinity (Version 1.9.3., Serif, United Kingdom).

## Results

### Xenopus and axolotl larvae show a comparable developmental pattern

**Figure 3:**
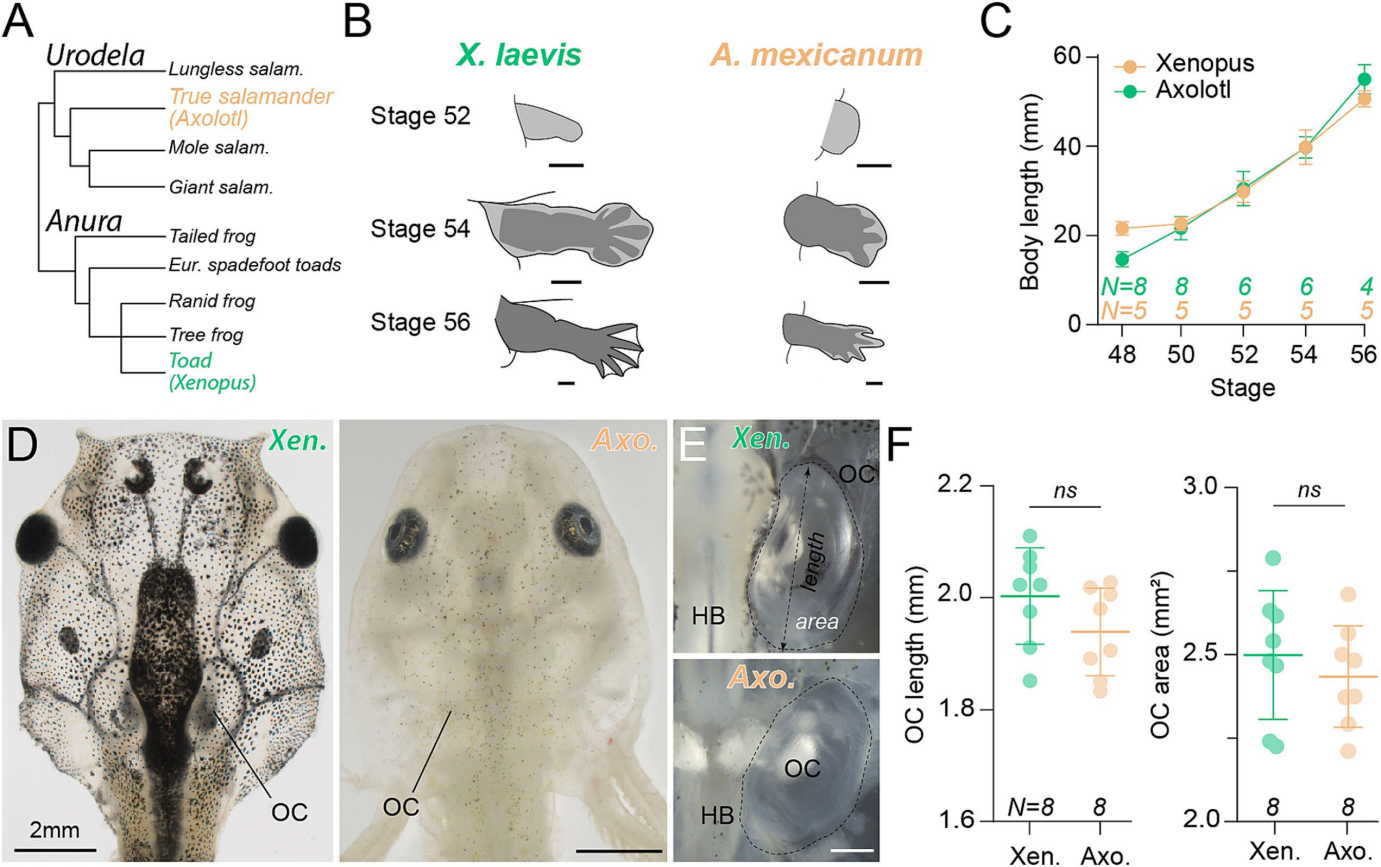
Developmental pattern of *Xenopus* and axolotl larvae. **(A)** Phylogenetic tree of anurans and urodeles with a common ancestor about 320 million years ago. **(B)** Limb bud morphology in both *Xenopus* and axolotl at stages 52, 54, and 56 (scale bar = 50 μm). **(C)** Growth curve measured as total body length in mm across stages 48 (*N* = 8 Xen.; 5 Axo.), 50 (*N* = 8 Xen.; 5 Axo.), 52 (*N* = 6 Xen; 5 Axo.), 54 (*n* = 6 Xen; 5 Axo.), and 56 (*N* = 4 Xen; 5 Axo.). **(D)** Representative images of a *Xenopus laevis* (Xen., left) and axolotl (Axo., right) larval head at stage 54. **(E)** Magnification of the hindbrain (HB) and otic capsule (OC) in larval stage 54 *Xenopus* (top) and axolotl (bottom). Dotted line indicates the selected region for otic capsule area calculations, dotted arrowhead lines indicate otic capsule length calculation axis. **(F)** Dot plot showing the mean ± SD for otic capsule length (left, *N* = 8 Xen.; 8 Axo.) and area (right, *N* = 8 Xen.; 8 Axo.) measured as indicated in **(E)**. Statistical significance was calculated by the Mann-Whitney *U*-test, two-tailed, *p* = 0.1049 and *p* = 0.5737, respectively. Scale bar in B = 50 μm; D = 2 mm; E = 0.5 mm.

We assessed swim kinematics and eye movements of the anuran *Xenopus laevis* and the urodele *Ambystoma mexicanum* (axolotl), which share a common Ichthyostegid ancestor in the middle Paleozoic era [(46); Figure 3A]. While both species can be maintained under very similar conditions in a laboratory environment, our initial aim was to verify the comparability of both species concerning anatomical features and developmental timepoints (Figures 3B–F). In both species, developmental stage identification is based on morphological features such as limb growth during ontogeny [Figure 3B; (33, 34)]. Globally, both species demonstrated a similar developmental temporal course with a comparable time range for a given stage, with a rapid growth until stage 40 followed by a slower late development (Supplementary Figure 1). In our laboratory conditions (18–20°C), it took at least 40 days in both species to reach stage 54, the main stage used in this study (Supplementary Figure 1). From stage 52 to stage 56, the end of the pre-metamorphic period, *Xenopus* and axolotl showed a similar hindlimb bud growth and foot finger differentiation (Figure 3B) as well as a similar body size growth over time with no significant difference across the examined stages (Figure 3C) and demonstrated a highly comparable developmental pattern along the larval period. Otic capsule length and area were not significantly different between both species at a given stage (Figures 3D–F), validating that variations in inner ear endorgan morphology were not due to general size differences of the head or otic capsule size themselves. Thus, the comparable developmental pattern and the common aquatic environment shared by the two species minimize the impact of significant behavioral variability that could mask the potential effect of the semicircular canal geometry on vestibular sensitivity and potentially on the swimming activity between *Xenopus* and axolotl.

### *Xenopus* exhibit larger vestibulo-ocular-induced eye movements than axolotl

*Xenopus* tadpoles perform a robust angular vestibulo-ocular reflex (aVOR) after reaching developmental stage 53 (16, 30, 40). Therefore, we directly assessed the visuo-vestibular ocular reflexes in whole head *in vitro* preparations of *Xenopus* and axolotl at larval stage 54 to compare the dynamics of gaze-stabilizing behavior of both species. In complete darkness, stimulation of the horizontal semicircular canal during sinusoidal head rotations at 0.5 Hz with a peak velocity of ±31.4°/s (±10° positional excursion), evoked robust reflexive compensatory eye movements in *Xenopus* which were oppositely directed to the stimulus position (Figures 1A,B, green), with an average gain (eye motion amplitude/stimulus amplitude) of 0.29 ± 0.1 (Figure 1C, green; mean ± SD, *N* = 6). They also exhibited a phase lead of −54.3° ± 12.34° (Figure 1D, green; r = 0.98) relative to the peak stimulus position at 0°. This aVOR response was similar to previous findings reported in larval *Xenopus* (16, 30, 40, 47). Inversely, head rotation did not evoke any vestibulo-ocular responses in larval salamander before stage 54 (Figure 1A). At stage 54, axolotl larvae subjected to the same stimulus exhibited comparatively poor aVOR performances (Figures 1A,B, orange) with an average gain of only 0.05 ± 0.02 (Figure 1C, orange; mean ± SD, *N* = 10). Apart from a significantly reduced gain (*p* < 0.0001, Mann–Whitney *U*-test, two-tailed), the aVOR exhibited a phase-lag *re* stimulus (p < 0.0001, Watson-Williams F-test) by 47.06° ± 24.48° (Figure 1D, orange; r = 0.91). Comparison of gain values across developmental stages revealed that in both species the gain follows a linear increase, albeit with a major difference in the onset of the aVOR. While semicircular canal-driven eye motions become functional at stage 49 (gain ≥ 0.12) in *Xenopus* (16), this lower threshold was equivalent to our recorded gain in axolotl at stage 54, indicating that the aVOR in this species only becomes sustainable at stage 56 (0.15 ± 0.04, mean ± SD; Figure 1E, orange). However, the aVOR performed in axolotl at stage 56 remained much lower than in *Xenopus* at the same developmental stage (Figure 1E).

The OKR works in synergy with the VOR, as it detects preferentially slow visual motion, and thus lower frequencies than the VOR. It also serves as a feedback loop and cooperatively both reflexes ensure appropriate gaze stabilization (48). Consequently, the differences in VOR responses between the two species could be related to a weaker visual capacity and/or late maturation of the visual system in axolotl. Therefore, we next investigated the potential contribution of the OKR either conjointly activated with the VOR (Figures 1F–H, Supplementary Figures 2A,B) or in isolation (Figures 1I–L, Supplementary Figure 2B). We tested whether perception of the visual field could improve the VOR in axolotl. For the activation of the aVOR under visual conditions (VOR in light in Figure 1), animals of both species were subjected to horizontal sinusoidal head rotation at 0.5 Hz (±31.4°/s) in front of a world-stationary black and white striped pattern (Figures 1F–H). Under these conditions, *Xenopus* tadpoles exhibited VOR compensatory eye motion profiles with a higher gain of 0.32 ± 0.08 (Figures 1F,G, green, mean ± SD, *N* = 6) than the VOR or the OKR alone (Figure 1I), and exhibited an increased phase lead *re* stimulus (−29.4° ± 18.2°; Figure 1H, green, r = 0.96) compared to the vestibular-only condition. However, the response was still more phase led than during OKR stimulation alone (Figure 1L). For axolotl, activation of the VOR under visual conditions resulted in gains that were slightly higher than those under VOR-only conditions but lower than under OKR conditions (Figures 1F,G; *p* = 0.002, Wilcoxon matched-pairs signed rank test). Accordingly, under any conditions including vestibular stimulation, axolotl eye motion was smaller than observed in *Xenopus* (*p* = 0.0002, Mann–Whitney *U*-test, two-tailed) with an average gain of 0.14 ± 0.06 (Figure 1G, orange, mean± SD, *N* = 10). Like in the dark, the VOR in light gain increased slightly in stage 56 axolotl (0.219 ± 0.05, mean ± SD N = 5; Supplementary Figure 2B) but remained lower than in *Xenopus*. Quantifications of phase relative to peak stimulus position were similar to exclusive VOR stimulation, leading by 41.52° ± 18.62° *re* stimulus (Figure 1H, orange, r = 0.95). Sinusoidal rotation of a black and white vertical striped pattern at 0.1 Hz and ±6.28 °/s (±10°) resulted in stimulus-following optokinetic motor responses for both species. Conversely to VOR responses, the OKR was stronger in axolotl at a gain of 0.25 ± 0.09 (mean ± SD, *p* = 0.011, Mann–Whitney *U*-test, two-tailed) compared to *Xenopus* at 0.14 ± 0.04 [Figures 1I–K, green, *N* = 6, (29, 41)]. In both cases, the average eye positions faithfully followed the stimulus (Figures 1I,J) and were nearly in phase with the peak stimulus position at 13.4° ± 10.1° (Figure 1L, green, r = 0.99) for *Xenopus* and at 6.03° ± 13.9° (Figure 1L, orange; mean ± SD, r = 0.97) for axolotl. Pre-motor vestibulo-ocular and motor extraocular neuronal relays are common to both OKR and VOR pathways (49). Consequently, the higher optokinetic response observed in axolotl compared to *Xenopus* demonstrates that the weak aVOR exhibited by the salamander larvae at stage 54 is not due to a delayed maturation in ocular motor circuits responsible for horizontal eye movements but rather might come for a deficit at the sensory level. Also, the comparable OKR response observed in axolotl from stage 54 to 56 (Figure 1G and Supplementary Figure 2B) suggest that the differences observed in the salamander VOR is unlikely related to a substantial maturation of the visual system during that period.

### Axolotl exhibit a reduced semicircular canal size with a distinct morphology compare to *Xenopus*

The observed inferior execution of the VOR in axolotl compared to *Xenopus* larvae prompted us to quantify the morphology of the horizontal semicircular canal as the potential cause for the weak and delayed aVOR onset in the larval salamander. Canal geometry is critical for the endolymph flow dynamics and consequently for the capacity of semicircular canals to detect angular head accelerations (14). The rotational-driven endolymph flow deflects bundle cilia in the cupula that activate the hair cells. Therefore, the flow-enabling biophysical parameters, the initial endolymph velocity, the response latency but most importantly the maximum endolymph displacement, are often considered as a measure of vestibular sensitivity (9). Several morphological criteria like the canal length, circuit and lumen radii, the duct cross-section area and the ampulla morphology, have been identified to play a role in the endolymph flow kinetics (9, 10, 50).

**Figure 4:**
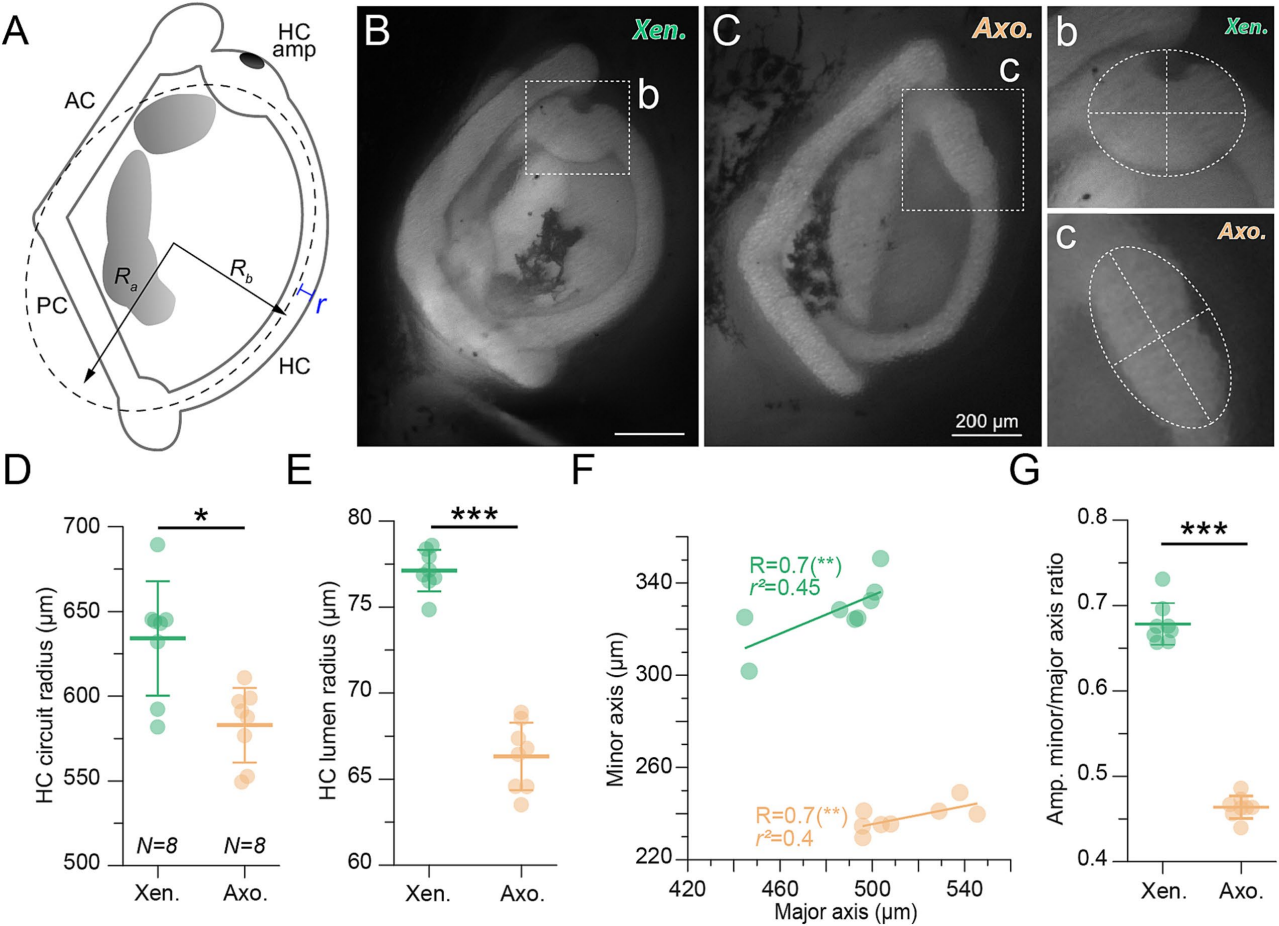
Two-dimensional horizontal semicircular canal morphology. **(A)** Top view schematic of the right semicircular canal spatial orientation showing an ellipse fitted (dotted line) to measure the circuit radius (calculated from the R_a_ and R_b_ ellipse radii) and the lumen radius (yellow “*r*”). **(B,C)** Representative examples of stage 54 *Xenopus* (Xen.) and axolotl (axo.) labyrinth injected with tetramethylrhodamine dextran dye; (b, c) magnifications of the ampulla of the horizontal semicircular canals shown in **B,C** with an ellipse fit (white dotted line) to measure the ampulla size (see **F** and **G**). **(D,E)** Circuit **(D)** and lumen radii **(E)** of the horizontal canal (HC) depicted as mean ± SD in *Xenopus* (green, *N* = 8) and axolotl (orange, *N* = 8); *p* = 0.3282, *p* = 0.0002, respectively, Mann Whitney U-test, two-tailed. **(F)** Correlation of the major and minor axis of the ellipse fitted on the ampulla (see b, c) in *Xenopus* (green) and axolotl (orange). **(G)** Ratio of the major and minor axis of the HC canal depicted as mean ± SD. ***p < 0.001; Mann-Whitney *U*-test. *R_a_* major axis, *R_b_* minor axis, *r* lumen radius. HC, PC, AC: horizontal, posterior, anterior canal, respectively; HC amp: HC ampulla. Scale bar in B and C = 200 μm.

**Figure 5:**
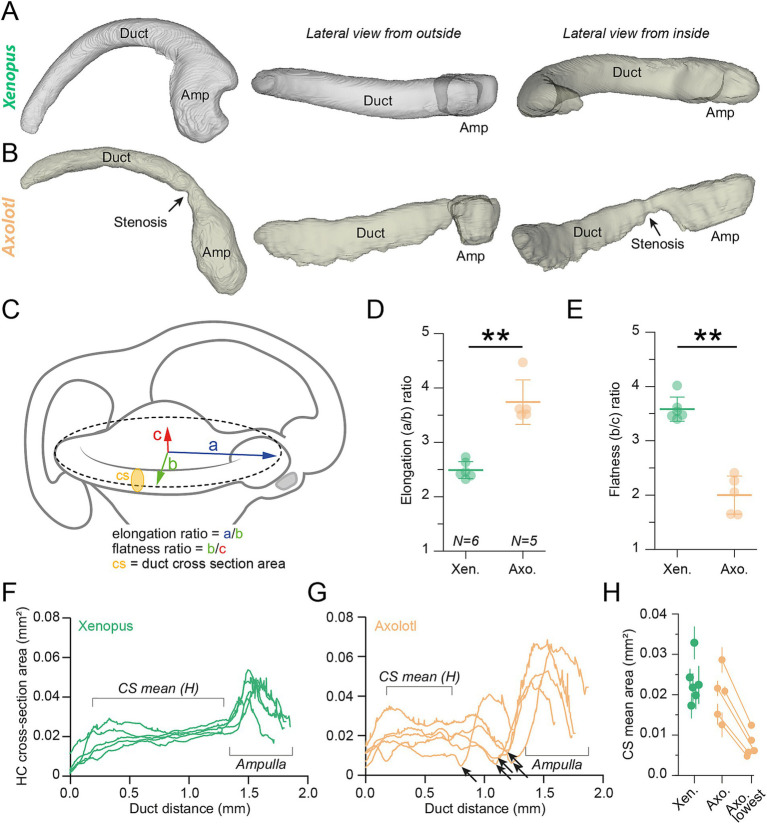
Three-dimensional horizontal semicircular canal morphology. **(A,B)** Representative reconstructed views of the 3D morphology of the horizontal canal in *Xenopus* (top row) and axolotl (bottom row) at stage 54. The black arrow in the canal views of the axolotl duct points to the stenosis of semicircular duct end of the ampulla (see also panel **F–H**). **(C)** 3D Schematic of the vestibular canal system showing metrics for the 3D spatial measurements. Three oriented vectors are extracted from the 3D reconstruction; the “a” (blue) and “b” (green) vectors correspond to R_a_ and R_b_ of the fitted ellipse (like in this panel), the “c” (red) vector corresponds to the vertical elevation component from the lowest to the highest detected limits of the canal. The yellow disk represents the duct cross section (cs) area measured all along the duct (see **F,G**). **(D,E)** Elongation (**D**, a/b) and Flatness (**E**, b/c) ratios of the horizontal canal depicted as mean ± SD in *Xenopus* (green, *N* = 6) and axolotl (orange, *N* = 5); *p* = 0.0043, *p* = 0.0043, respectively, Mann Whitney U-test, two-tailed. **(F,G)** Cross-section areas along the horizontal canal duct length in *Xenopus* (green, *N* = 5 in **F**) and axolotl (orange, *N* = 5 in **G**); note the narrow canal lumen (stenosis) prior to the canal duct end of the ampulla in axolotl indicated by a black arrow. **(H)** Mean (± SD) of the cross section (CS) area calculated along the duct region indicated in panel **F,G** compared to the lowest cross section (Axo. lowest) area found at the duct stenosis (black arrow in panel **G**) in axolotl.

In particular, canal circuit (R, Figure 4A) and duct (r, Figure 4A) radii appear to be the two major morphological components determining the onset of the aVOR during vertebrate development [(15, 16); for theoretical aspects see Muller (10)]. The late aVOR appearance in larval axolotl life (at stage 54, Figure 1E), compared to *Xenopus*, led to consider the same developmental mechanism than previously described to explain the onset of the aVOR in *Xenopus* stage 49 (16). Before stage 54, larval axolotl exhibit horizontal semicircular canal size insufficient to detect angular head motion. Only at stage 54 or later canal circuit and duct radii reach the same critical size threshold to trigger a functional VOR response, comparable to stage 49 in *Xenopus*. Therefore, we examined the canal circuit and lumen radii in both species at stage 54 and 56. A fluorescent tracer (see methods section) was injected into the *common crus* of the anterior and posterior canal allowing for visualization of the canal morphology in 2- and 3-dimensions following fluorescence microscopy (Figures 4, 5). Fitting an ellipse on the horizontal semicircular canal imaged from dorsal view (Figure 4A) revealed that the circuit radius was smaller in axolotl (582.91 ± 22.05 μm; *N = 8*) than in *Xenopus* (634.09 ± 33.73 μm; *N = 8*; Figure 4D, *p* = 0.0104, Mann Whitney U-test, two-tailed). Moreover, the canal radius (r) was also significantly smaller in axolotl (66.32 ± 1.95 μm; 77.11 ± 1.2 μm; Figure 4E, *p* = 0.0002, Mann Whitney U-test, two-tailed). As hypothesized, canal radii measurements of axolotl at stage 54 were comparable to values found in *Xenopus* at stage 49 [R ~ 600 μm, r ~ 70 μm; (16)], indicating that semicircular canal morphology limits vestibular performance prior to this stage. Concomitantly to the increase of the aVOR gain (see Figure 1E), axolotl also demonstrated larger circuit and lumen radii at stage 56 but still lower than in *Xenopus* at the same stage, especially for the lumen radius (Supplementary Figure 3A). Another difference observed between both species was the shape of the ampulla, the enlarged region at one extremity of each canal, which houses the hair cells (Figure 4A). A wide and less round ampulla limits endolymph flow detection by hair cells and thus canal sensitivity (9, 13, 14). Quantification of ampulla size and roundness, by calculating the linear regression and the ratio between the major and minor axis (Figures 4B,C), indeed revealed a more elliptical ampulla with a larger length for axolotl (Figures 4F,G, orange, *N* = 8) than for *Xenopus* (Figures 4F,G, green, *N* = 8; a ratio of 1 in the Y axis corresponds to a perfect circle), which likely contributes to comparatively lower canal sensitivity in axolotl.

For a more comprehensive morphological distinction, we reconstructed 3D models of the entire horizontal semicircular canal from confocal image stacks (see methods; Figures 4B,C). These revealed significant differences in the overall shape of *Xenopus* and axolotl horizontal canals (Figures 5A,B respectively). Ellipsoid fitting on the HC canal in 3 dimensions allowed measuring a true semi-major (blue axis “a” in Figure 5C) and semi-minor axis (green axis “b” in Figure 5C), allowing to measure the elongation of the canal as the elongation ratio (major semi-axis (a)/minor semi-axis(b), the lower the a/b ratio, the rounder the canal, Figure 5D). Additionally, the depth axis (red semi-axis “c” in Figure 5C) which shows the vertical projection of the canal curvature, was measured to calculate the flatness ratio (Figure 5E) as the minor semi-axis (b) / vertical projection (c), showing how planar/straightly oriented along the horizontal axis the canal is (the higher b/c ratio, the more planar the canal). Cupula hair cells are unidirectional which means that the optimal canal sensitivity would be obtained when the endolymph flow deflects the hair cell bundles towards the kinocilium. Consequently, a longer canal with a regular curvature coplanar to the horizontal head rotation would maximize the endolymph flow displacement by limiting the disturbances due to more variable frictions tangentially to the duct wall and/or to non-planar duct orientations (9, 50). A low elongation ratio and a high flatness ratio will provide a canal with a regular curvature and coplanar to the horizontal head plane. Such a morphology should help to produce a more regular and smooth endolymph flow, with minimum disturbance. Inversely, a high elongation ratio coupled with a low flatness ratio demonstrates a canal duct with a more irregular curvature and a non-planar orientation to the horizontal head plane, potentially enhancing a more perturbated endolymph flow. Elongation and flatness ratio calculations showed that HC canals in axolotl were less curved (Figure 5D, *p* = 0.0043, Mann–Whitney *U*-test, two-tailed) and less flat (Figure 5E, *p* = 0.0043, Mann–Whitney *U*-test, two-tailed) than in *Xenopus*, both of which likely restrict endolymph motion and further contribute to reduced sensitivity. In the case of these two species the consequence of a reduced curvature in axolotl led to a smaller duct length (*Xenopus* duct length = 1.4 ± 0.15 mm; axolotl duct length = 1.07 ± 0.17 mm; mean ± SD; see Figures 5F,G for individual; see Supplementary Figure 3C for stage 56), a parameter known to affect the vestibular sensitivity, as explained above. Comparable values of elongation and flatness ratio were found for the two species at stage 56, showing that the curvature and planar organization did not changed significantly during that period (Supplementary Figure 3B). Finally, plotting of the canal cross section (yellow disk “cs” in Figure 5C) across the entire length of the canal (Figures 5F,G) revealed a constricted area/stenosis of the duct just at the entrance of the ampulla in axolotl (Figures 5B–G, arrow) which was absent in *Xenopus*. At the stenosis location, the lowest cross section area was less than half the mean cross section area calculated all along the duct (Figure 5H; the CS mean area was calculated as indicated in Figures 5F,G), and below the standard deviation calculated in each animal (Figure 5H). This stenosis was also observed in axolotl at stage 56, confirming that this is a specific morphological characteristic of the axolotl horizontal canal that is not present in *Xenopus* (Supplementary Figure 3C). Overall, results from 2D and 3D analyses demonstrated that *Xenopus* and axolotl exhibited horizontal semicircular canals with significantly different morphological characteristics. Such distinctions in canal morphology suggested, according to previous theoretical knowledge [for a review see Lambert and Bacqué-Cazenave (14)], a potentially restrained biomechanical activation of hair cells by endolymph flow in axolotl compared to *Xenopus* larva.

### Swimming activity is different between *Xenopus* and axolotl larva

We recorded the swimming activity of freely moving animals in a circular dish from the top (Figure 2A) and tracked the x-y position of body parts across time using SLEAP (36). This allowed us to extract several locomotor kinematic parameters like the swim distance, speed, bout length, tail deflection velocity, and angular head acceleration across time. This revealed a different locomotor activity between the two species. *Xenopus* swam rather continuously at a relatively constant speed (see examples in Figures 2B,D), whereas axolotl exhibited interspersed, short bouts of locomotion with high speeds followed by a short passive glide until stationary (Figures 2C,E). Active locomotion was identified as times where animals moved while also deflecting their tail (Figures 2F,G, black bars) and quantified for each animal (Figure 2H). *Xenopus* spent significantly more time locomoting than axolotl, with 28.8 s as opposed to 10.7 s on average (per 60 s of recording) (Figure 2H *p* = 0.0188, Mann Whitney U-test, two-tailed), as previously reported (51). While *Xenopus* also demonstrated more swim bouts in general (see inset in Figure 2I), we also investigated if majority of swimming occurred in singular long, or multiple short bouts. Each swim bout was weighted relative to their contribution to total swimming, i.e., a 50 s swim bout counted 50 times more than a 1 s bout. This showed swimming in *Xenopus* is mostly contained in longer swim bouts of around 7 s (6.98 s ± 3.74 s; mean ±SEM; N = 13, n = 203 bouts), while axolotl swimming occurs mostly in bouts of less than a 1 s (0.92 s ± 0.08 s; mean ±SEM; N = 12 animals, n = 67 bouts) duration (Figure 2I, *p* < 0.0001, Kolmogorov–Smirnov test). Thus, *Xenopus* spent more time actively locomoting in longer swim events, while axolotl spent less time locomoting and did so in short, individual bouts. As active locomotion necessitates gaze stabilization, this may indicate a stronger need for *Xenopus* to maintain a stable gaze. Perhaps more important than the amount of motion is its characteristics to be compensated, and we thus looked next at the main parameter detected by vestibular endorgans. During undulatory swimming, the head rotates mainly in the horizontal plane, which is mostly picked up by the horizontal semicircular canals as angular acceleration (31, 52). Quantification of the angular head acceleration profiles were found to be different (Figure 2J, *p* = 0.012, Kolmogorov–Smirnov test), even if both species exhibited an acceleration peak quite close (192.32°/s^2^ in *Xenopus* vs. 202.01°/s^2^ in axolotl; Figure 2J, left Y axis). Indeed, swimming activity in axolotl led to a reduced amount of head angular acceleration (above 250°/s^2^) compared to *Xenopus*, as indicated by the cumulative density (Figure 2J, right Y axis). Altogether, these results suggest that, in laboratory experimental conditions, larval *Xenopus* exhibit more and longer locomotor events, during which they are exposed to a wider range of head accelerations, albeit at a lower peak velocity. Vice versa, axolotl at a comparable developmental stage locomote less, and do so with short, less variable movements with higher peak accelerations. Based on previous descriptions of locomotor styles of these animals, this is very likely to translate to naturalistic conditions (51).

**Figure 6:**
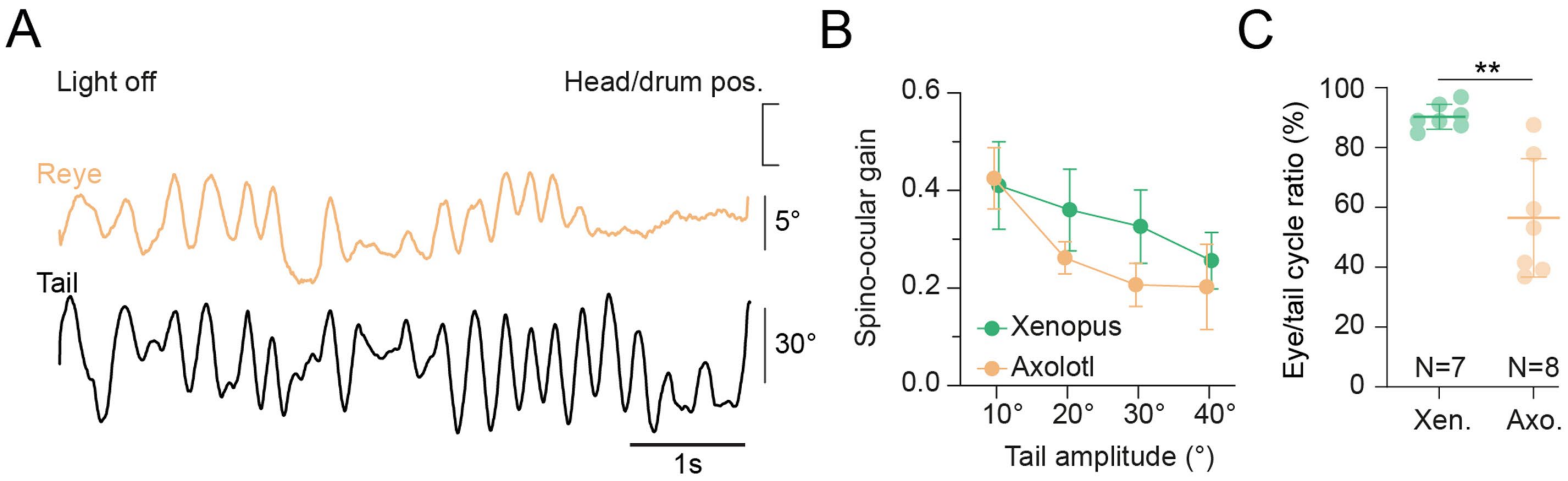
Locomotor-induced gaze-stabilizing eye movements at stage 54. **(A)** Representative compensatory eye movements evoked by the locomotor spino-ocular coupling during head-fixed swimming in the dark. **(B)** Average gain (eye motion amplitude/tail amplitude; mean ± SD) vs. tail amplitude for locomotor-induced eye movements between stage 54 *Xenopus* (*N* = 7) and axolotl (*N* = 8) respectively. **(C)** Averaged (± SD) eye/tail cycle ratio (proportion of eye movement related to tail movement). **p < 0.001; Mann-Whitney *U*-test.

During rhythmic locomotion, reflexive gaze stabilizing eye movements as measured above are additionally complemented by an efference copy feedforward signal from spinal central pattern generators, and accordingly, these could provide a further source to compensate for the lack of vestibular input during swimming [Lambert et al. (76) for review see Straka et al. (53)]. However, the maturation and efficiency of locomotor-induced gaze stabilizing eye movements rely on the onset and maturation of semicircular canal sensitivity during larval development in *Xenopus* (30). Taking into consideration this conjoint maturation, we next quantified locomotor-induced ocular activity in larval axolotl in comparison to *Xenopus* to see whether vestibular signalling is sufficient to enable efference-copy driven eye motions, and if these could drive compensatory eye motions during locomotion (Figure 6). In the absence of any visuo-vestibular sensory input (see methods), undulatory swimming in semi-intact *in vitro* head fixed preparations of stage 54 axolotl produced conjugated eye movements, phase-coupled to the tail movement but in the opposite direction (Figure 6A) comparable to those previously described in larval stage 54 *Xenopus* (30, 54). In both species the spino-ocular gain gradually decreased with large tail amplitudes but with a more pronounced tendency in axolotl than in *Xenopus* even if the difference was not significant (Figure 6B; *p* = 0.43, simple linear regression). However, the spino-ocular motor coupling was much less efficient to produce compensatory eye movements correlated to each tail cycle in axolotl than in *Xenopus*, with an eye/tail cycle ratio of 56.49% ± 19.76% in axolotl and 90.31% ± 4.15% in *Xenopus* (Figure 6C, mean ± SD, *p* = 0.0023, Mann–Whitney *U*-test, two-tailed).

### Correlative relationship between canal size, vestibular sensitivity, and locomotion

**Figure 7:**
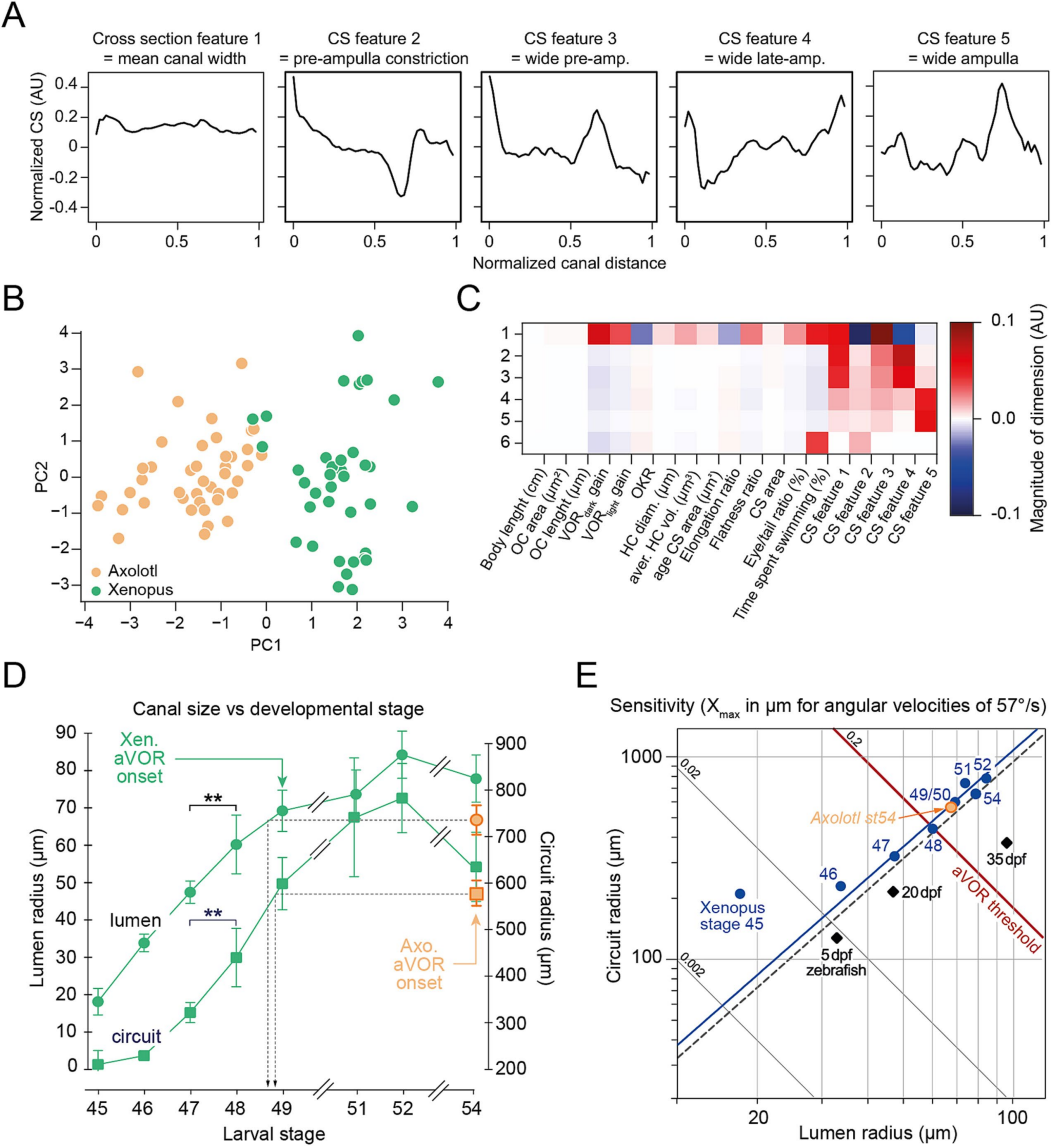
Correlative relationship between canal geometrical, vestibular and locomotor parameters. **(A)** Horizontal canal lumen radius (left Y axis) and circuit radius (right Y axis) measured (mean ± SD) in larval *Xenopus* (Xen.) by Lambert et al. (16) updated with radii measurements in larval *Xenopus* and axolotl (axo.) at larval stage 54 provided by this study. **(B)** Mean circuit radius versus lumen radius relative to the canal sensitivity (X_max_, oblique lines) originally published in Lambert et al. (16) and updated with canal radii values from *Xenopus* and axolotl larval stage 54. **(C)** Cross-section (CS) features 1–5 (left to right) as extracted by PCA on interpolated cross-section profiles of all animals. CS feature 1 represents the mean difference in cross section area. CS feature 2 represents a strong constriction at the approximate start of the ampulla. CS feature 3 represents a wide ampulla opening. CS feature 4 represents a steady increase in cross-section area. CS feature 5 represents a wide mid-Ampulla. Overall the first 5 PCs account for 98% of the variance in the cross-section data. **(D)** Measurement vectors (MVs, orange: axolotl, green: *Xenopus*) of all animals projected onto first and second principal component (arbitrary units). The variables going into the MVs were body length [cm], OC area [μm^2^], OC length [cm], VOR D gain, VOR L gain, OKR gain, HC Diameter [μm], HC Volume [μm^3^], average cross section area [μm^2^], elongation ratio, flatness ratio, eye/tail ratio (%), time spent swimming (%), CS feature 1, CS feature 2, CS feature 3, CS feature 4, CS feature 5. MVs were obtained by resampling the results of each measurement type for each species within all animals for which the respective measurement was missing. MVs were normalized and centered around zero before PCA (see Methods). **(E)** Heat map of PC vector values scaled by attributed variance. Red: positive correlation of PC with variable. Blue: negative correlation of PC with variable.

To analyze canal morphology in an unbiased way, unsupervised canal features were extracted from the raw cross section profiles by taking the first 5 principal components (Figure 7A and Supplementary Figure 4). The first cross section feature is mostly a positive constant along the canal, reflecting the average difference in cross section between axolotl and *Xenopus*. Cross section feature #2 describes and quantifies the sharp constriction/stenosis at the start of the ampulla, while being mostly flat otherwise. Cross section features #3–5 also mostly represent shape factors of the ampulla, indicating different exact positions of maximum width. To test the hypothesis of a functional correlative relationship among the various variables describing canal morphology, vestibular sensitivity and locomotive behavior and illustrate the relative contributions of them, all the data presented in this work was compiled into a comprehensive dataset by a resampling approach, giving each animal a full feature vector of 19 different measures (see Methods, Figure 7D and Supplementary Figure 4). axolotl and *Xenopus* specimens are clearly and robustly separated as clusters by the first principal component of this comprehensive dataset, but not the second (Figure 7B and Supplementary Figure 4A) or later components (Supplementary Figure 4A). This shows that the overall variance among all these variables is larger between the species than within each individual species, and that the mean difference between them is well described by a single feature vector. Inspecting the individual components scaled by the amount of variance they explain (Figure 7E; Supplementary Figure 4B), reveals that body length, OC area and OC length do not contribute anything to the first component and thus do not covary significantly with any of the other variables and between species. VOR gain, canal width, flatness ratio, a wide pre-ampulla and time spent swimming covary positively in the first principal component, while elongation ratio and a tight pre-ampulla covary strongly negatively (Figure 7E and Supplementary Figure 4B). Interestingly the pre-ampulla constriction and widening are the strongest negative and positive values, respectively, in the first principal component, meaning they contribute the most to the difference between axolotl and *Xenopus* and have the strongest covariance with the behavioral variables VOR gain and time spent swimming. Thus, the pre-Ampulla constriction seems to be the most relevant and determining functional correlate of axolotl’s behavioral phenotype differences to *Xenopus*, followed by the elongation and flatness ratios.

## Discussion

The results of this paper provide evidence for the functional correlation of semicircular canal morphology which constrains head motion detection, thereby restricting vestibular based gaze stabilization. We further show that such restrictions in vestibular sensitivity go hand in hand with differences in locomotor kinematics. While the data indicate that slight adaptations to different behavioral niches and phenotypes are abundant throughout various physiological characteristics and variables, they highlight the morphology of the ampulla as a main target for potential evolutionary adaptations of vestibular processing. All together, these results suggest that axolotl exhibit some key differences compared to *Xenopus* in locomotor activity that might be related to their weaker vestibular sensitivity induced by their canal geometry, thereby being less efficient to detect head motion.

### Semicircular canal morphology is an important ontogenetic determinant for vestibular sensitivity

In brief, biomechanical models of semicircular canals established that the maximum endolymph displacement (X_max_) in the canal is a direct measure for the canal sensitivity (10). This endolymph displacement is linearly dependent of both the circuit (R) and lumen (r) canal radii [X_max_ = Cst x R x r^2^ where “Cst” is a constant factor integrating several endolymph fluid parameters defined by Muller (10)]. In addition, theoretical studies propose several other morphological parameters as influencing the endolymph displacement into the canal: the variation of the canal radius along the duct, the shape of the ampulla but also the plane orientation of the duct (9, 50). All these parameters interfere with the endolymph flow direction. Like hair cells are all oriented the same way in the cupula, any distortion in the fluid direction, induced by the canal morphology, will impair the optimal hair cell activation during head movement. According to these biomechanical studies, the variation in canal radius along with the more elliptical shape of the axolotl ampulla suggests that endolymph flow is less favorable to detect head rotations in the larval salamander than in the larval toad. Furthermore, the 3D analysis revealed a lower canal circularity, a substantial distortion from a single spatial plane, and a stenosis of the duct just near the entrance of the ampulla in axolotl. These features contribute to a perturbation of the endolymph flow within the canal, leading to a deceleration of the input fluid into the ampulla and, consequently, a reduction in hair cell activation (14). Accordingly, a canal with such geometrical parameters would be less efficient in detecting head acceleration during both active and passive motion. Our results showed that the minimum canal size necessary to trigger aVOR response in stage 49 *Xenopus* (16) was not reached before stage 54 in axolotl.

Furthermore, this comparative study between *Xenopus* and axolotl confirmed this biomechanical canal size limitation and seems to be a common feature in VOR ontogeny throughout vertebrates as initially demonstrated in larval *Xenopus* (16) but also described in larval teleost fish (15) and probably in miniaturized frog (20). Larval zebrafish demonstrated a very late angular VOR onset during the development, around 35dpf, compared to some other fish species like medaka [around 20dpf; (15)]. Although no quantitative study of the semicircular canal morphology has been performed on larval zebrafish so far, a previous qualitative anatomical description of inner ear endorgans suggests that these teleost species could demonstrate a canal morphology comparable to what we observed in larval *Xenopus* and axolotl prior to the VOR onset (55). To support this conclusion, axolotl and *Xenopus* canal radii measurements at stage 54 were added to previous data published in 2008 (Figures 7D,E). Figure 7A illustrates that axolotl stage 54 shared comparable circuit (orange square) and lumen (orange circle) radii size with *Xenopus* stage 49 [from figure 6E in Lambert et al. (16)]. At these two larval stages for salamander and toad species, respectively, the canal sensitivity (directly deriving from R and r as explained above) reached the threshold to elicit a functional angular VOR (Figure 7E), demonstrating that the canal size limitation is the common determining feature in the ontogeny of the angular VOR in aquatic species like amphibians and fishes and probably in a larger extent in vertebrates.

### Consequences for swimming performances

By detecting head movements, and thereby ensuring a stable posture which is required for efficient locomotion, vestibular endorgans contribute to an optimization of locomotor parameters (gait, speed, trajectory). Vestibular inputs are used in the necessary postural adjustments (1) but also influence directly the locomotor activity in spinal motor networks (56–59). Consequently, there is a strong likehood that vestibular sensitivity could affect, to some extent, the locomotor activity and modes across vertebrate species. Firstly, otolith inputs build a body-in-space postural reference frame, a crucial prerequisite to establish a stable locomotor activity as demonstrated in larval frog (60–62) and more recently in larval zebrafish (63, 64). Indeed, gravito-inertial sensory signals define the body orientation and passive displacement relative to the gravitational vector, two positional parameters critical to calculate up and down swimming trajectories in aquatic animals. Interestingly, in *Xenopus* first vestibular projections to the spinal cord appear at stage 35, concomitantly to the onset of free swimming behavior, a larval stage where only otolith endorgans are functional (65, 66). This ontogenetic relationship between otolith-driven vestibular pathways and posturo-locomotor behavior might also exist in larval fishes (64, 67, 68). Secondly, undulatory swimming in anguilliform aquatic animals produces rhythmic oscillations of the head, thereby activating lateral semicircular canals through the resultant angular rotations. This canal sensory signal could influence locomotion strategy in several ways. First, canal inputs might have a direct influence on the locomotor activity through vestibulospinal pathways, notably on the bout length. Recent findings demonstrate that horizontal semicircular canal activation is able to trigger a locomotor postural response in *Xenopus* tadpoles (56). In addition, developmental suppression of semicircular canals by hyaluronidase treatment impacts undulatory swimming activity in larval frog by reducing the swimming frequency and the tail deflection amplitude (30). In this case, swimming performances could be also indirectly influenced through the canal-induced tuning of the gravito-inertial control of locomotion. Indeed, canal inputs are known to tune the translational VOR, elicited by utricles, during the larval development (69, 70). A similar maturation tuning mechanism must also occur in otolith-driven vestibulospinal signals necessary to elicit a stable locomotor activity as demonstrated in larval fish. Therefore, we can hypothesize that a canal geometry less efficient to detect head angular motion, like in axolotl, would impair the developmental tuning of the otolith signal and consequently the otolith control of locomotion. Our kinematic analysis showed a notable difference in the swimming style between our two amphibian species. Axolotls exhibit distinct swimming events, with a high-velocity forward thrust/propulsion followed by a passive glide, commonly referred to as a bout. This swimming mode is in high contrast to *Xenopus*, which show an almost continuous locomotion as indicated by a larger percentage of time allocated to swimming throughout the recording session and much longer bouts. This type of swimming mode distinction has also been observed and characterized in two related fish species, zebrafish and Danionella cerebrum (71). Larval zebrafish demonstrated bouts of swimming whereas Danionella cerebrum exhibited continuous swimming sequences suggesting that a lifestyle-dependent phenotypic distinction of vestibular endorgans could exist in larval fish as described here in larval amphibians.

The discontinuous, bout-like swimming activity could be a locomotor adaptation in axolotl imposed by the low vestibular sensitivity due to the non-optimal semicircular canal morphology: less variable accelerations may reduce the error or mismatch signal between an ineffective capacity to detect head rotations and a weak angular VOR, while subsequent straightforward glides and overall shorter swimming events would reduce head deflections, minimizing the need for self-motion detection overall. Such reduced but fast locomotion in axolotl indeed fits with their ecological niche. Axolotls, even in juvenile stages, are sit-and-wait predators and are paedomorphic retaining an aquatic lifestyle throughout life (72). In contrast, the anuran *Xenopus laevis* tadpoles are filter feeders, almost continuously in motion in the surrounding water (73, 74). Previous investigations also observed less efficient swimming in adult axolotl compared to anuran tadpoles and most fishes and was speculated to be an adjustment to living in shallow lakes with dense vegetation (75). From this angle, reduced necessity for swimming may lead to less evolutionary pressure to rapidly develop functional inner ears, providing an alternative link between our morphological and behavioral data.

Altogether our findings reinforce the idea of an ecology-dependent relationship between semicircular canal morphology, locomotor, and vestibular functional capacity. However, a causative demonstration between lifestyle and vestibular performance is challenging to show. Nonetheless, it is clear that both vestibular sensation and locomotion require a co-adaptation, and this co-adaptation can be observed in many vertebrates’ taxa. On this purpose, aquatic species like fish and amphibians, appeared to be of particular interest to continue exploring this question along the line developed in the present study.

## Data Availability

The raw data supporting the conclusions of this article will be made available by the authors, without undue reservation.

## Footnotes

^1^ https://github.com/NeuroMorph-EPFL/NeuroMorph/wiki/Centerlines-and-Cross-Sections

## In memoriam

We dedicate this article to the memory of our mentor Prof. Dr. Hans Straka, who sadly and unexpectedly passed away in December 2022. He left a big hole in our research community and is greatly missed by all of us.

## Supplementary material

SUPPLEMENTARY FIGURE 1 Developmental time course of *Xenopus* and axolotl showing at which days post-fertilization larva reach a given morphological stage. The time course depends on the water temperature. The entire time course for *Xenopus laevis* is given on the Xenbase website for breeding condition at 23°C (https://www.xenbase.org/xenbase/anatomy/alldev.do). The dashed line time course for *Xenopus* is a relative time course from a batch of animals that were at stage 40 in the laboratory facility (University of Bordeaux) and raised at 18–20°C. The axolotl time course is based on observation made in the animal facility of the LMU at 20°C.

SUPPLEMENTARY FIGURE 2 **(A)** Representative example of compensatory eye movements evoked by the aVOR in light during horizontal sinusoidal rotation of the head/table (0.5 Hz; ± 31.4°/s) for one *Xenopus* and axolotl stage 54 larva, respectively. **(B)** Average gain and phase (mean ± SD) of the aVOR in light at 0.5 Hz (± 31.4°/s) in axolotl stage 56 (*N* = 5).

SUPPLEMENTARY FIGURE 3 **(A)** Circuit and lumen radii for horizontal semicircular canal (HC) in *Xenopus* (xen) and axolotl (axo) at stage 56. **(B)** Elongation and flatness ratio in *Xenopus* and axolotl stage 56. **(C)** Cross-section areas along the horizontal canal duct length in axolotl (*N* = 5); note the narrow canal lumen (stenosis) prior to the canal duct end of the ampulla indicated by a black arrow.

SUPPLEMENTARY FIGURE 4 **(A)** Expanded palette comparing the first three principal components of comprehensive semi-synthetic dataset. Diagonal: histograms of species datapoints along component. Off-diagonal: scatter plots of data in coordinates of pair-wise principal components. **(B)** Magnitudes of individual coordinates in PC1 across all 10,000 resamplings. Due to the arbitrary polarity of the PC1 vector, in some cases the coordinates are inverted. **(C)** Variance explained across all 10,000 resamplings for all principal components.
