# Supplementary figures and images for: Influence of semicircular canal morphology on the VOR and swimming activity in larval amphibians: a comparative study in Xenopus and axolotl
Source: Front Neurol. 2025 May 19;16:1564585. doi: 10.3389/fneur.2025.1564585 (PMC12127180; doi:10.3389/fneur.2025.1564585)

Schneider-Soupiadis et al.,  
Supplemanetal Figure 1

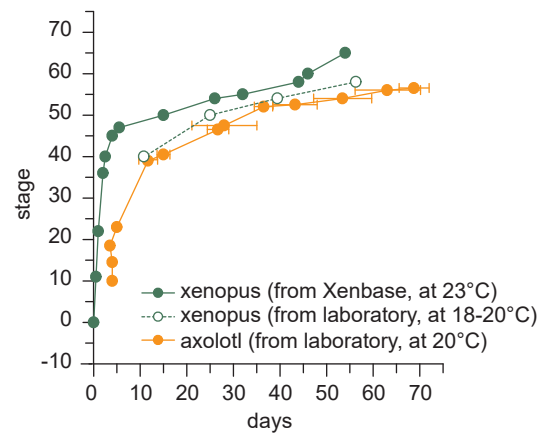

Supplement: Supplementary file 1 [file Data_Sheet_1.pdf]

A

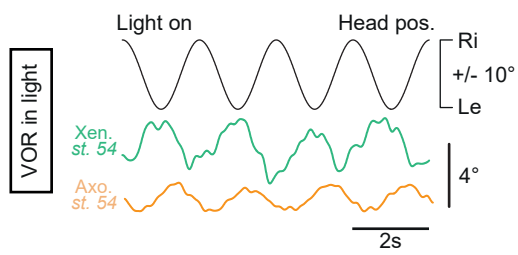

B

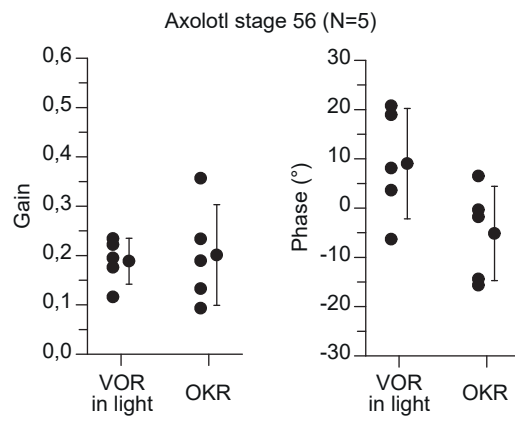

Supplement: Supplementary file 2 [file Data_Sheet_2.pdf]

A

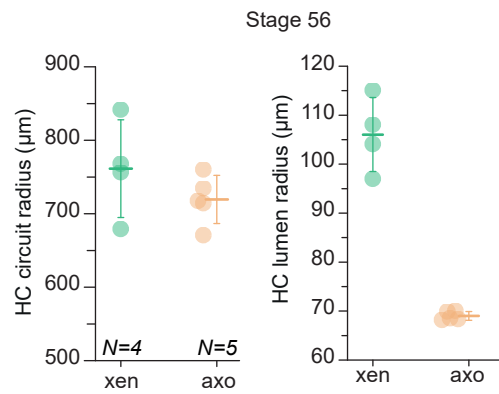

B

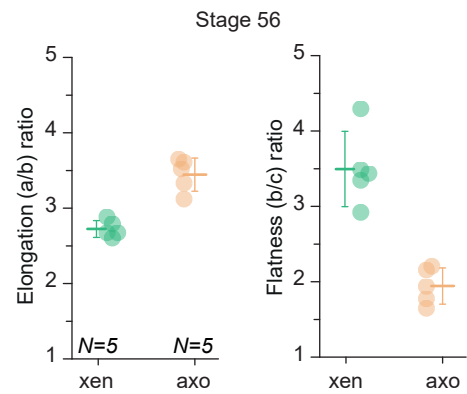

C

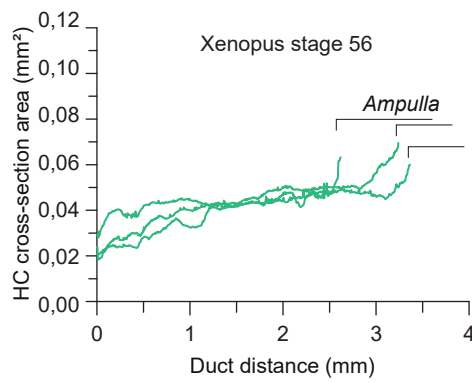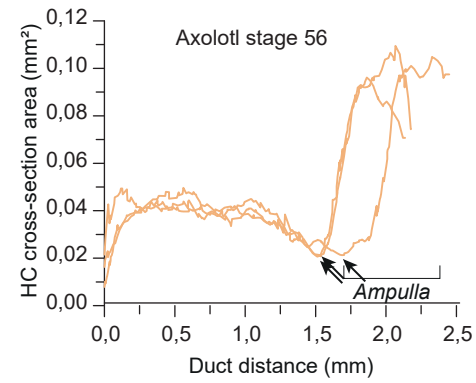

Supplement: Supplementary file 3 [file Data_Sheet_3.pdf]

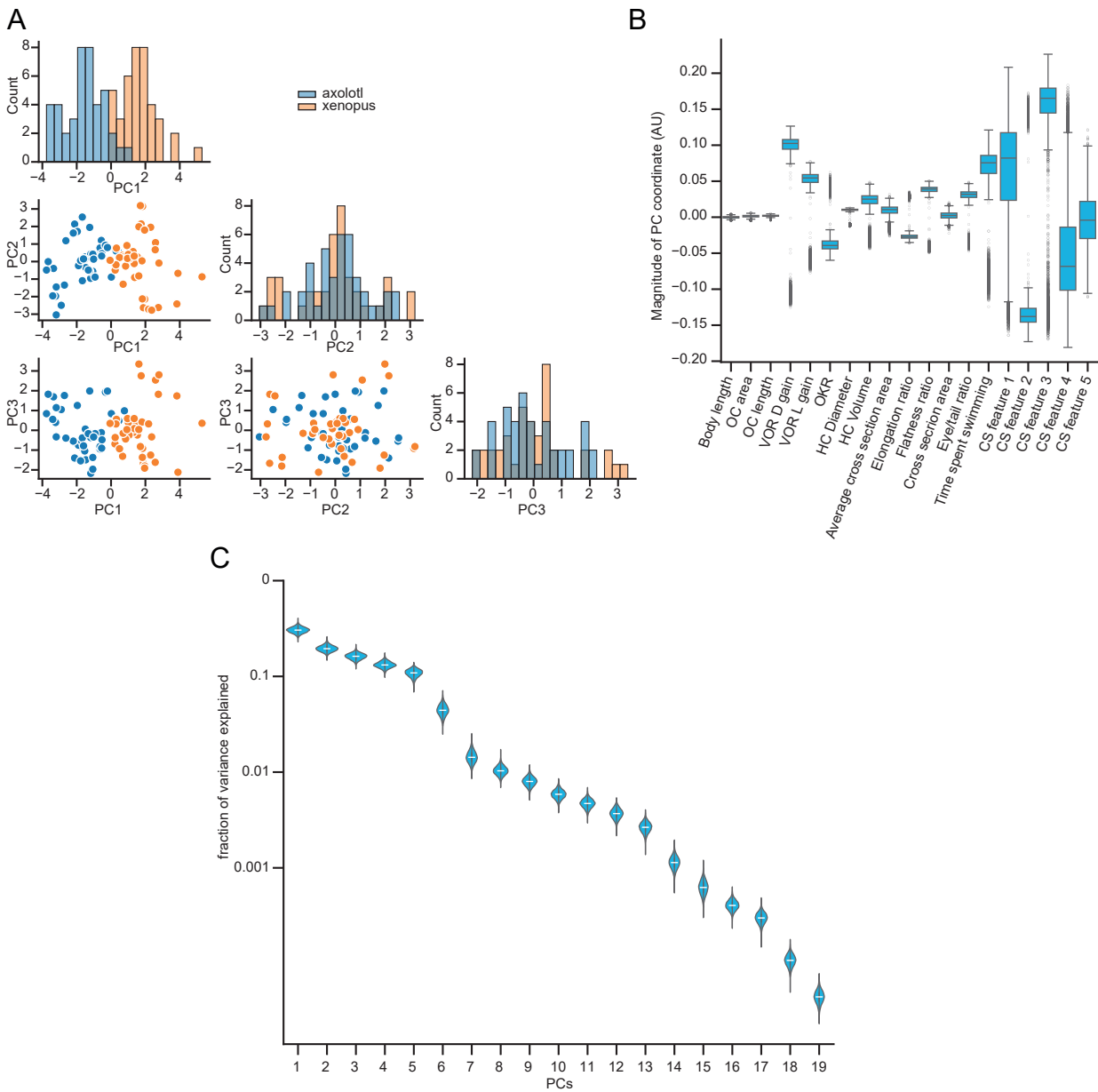

Supplement: Supplementary file 4 [file Data_Sheet_4.pdf]
